# miRNA-Dependent Translational Repression in the *Drosophila* Ovary

**DOI:** 10.1371/journal.pone.0004669

**Published:** 2009-03-02

**Authors:** John Reich, Mark J. Snee, Paul M. Macdonald

**Affiliations:** Section of Molecular Cell and Developmental Biology, Institute for Cell and Molecular Biology, The University of Texas at Austin, Austin, Texas, United States of America; UT MD Anderson Cancer Center, United States of America

## Abstract

**Background:**

The *Drosophila* ovary is a tissue rich in post-transcriptional regulation of gene expression. Many of the regulatory factors are proteins identified via genetic screens. The more recent discovery of microRNAs, which in other animals and tissues appear to regulate translation of a large fraction of all mRNAs, raised the possibility that they too might act during oogenesis. However, there has been no direct demonstration of microRNA-dependent translational repression in the ovary.

**Methodology/Principal Findings:**

Here, quantitative analyses of transcript and protein levels of transgenes with or without synthetic *miR-312* binding sites show that the binding sites do confer translational repression. This effect is dependent on the ability of the cells to produce microRNAs. By comparison with microRNA-dependent translational repression in other cell types, the regulated mRNAs and the protein factors that mediate repression were expected to be enriched in sponge bodies, subcellular structures with extensive similarities to the P bodies found in other cells. However, no such enrichment was observed.

**Conclusions/Significance:**

Our results reveal the variety of post-transcriptional regulatory mechanisms that operate in the *Drosophila* ovary, and have implications for the mechanisms of miRNA-dependent translational control used in the ovary.

## Introduction

Small regulatory RNAs act in a wide range of processes that contribute to control of gene expression. In eukaryotes, three classes of such RNAs have been characterized most extensively. Small interfering RNAs (siRNAs) mediate RNA interference or silencing, in which target mRNAs are degraded. microRNAs (miRNAs) are identical in structure to siRNAs, but have different origins and processing. miRNAs regulate protein accumulation from target mRNAs by a variety of mechanisms. Repeat associated small interfering RNAs (rasiRNAs) are synthesized through yet another pathway, and function in both chromatin organization and mRNA degradation. Each class of small RNA acts in conjunction with a protein complex consisting of an Argonaute family member and associated proteins: the RNA provides specificity through base pairing, either complete or incomplete, with targets, and the proteins act as effectors by various mechanisms that in most cases are not yet fully understood [1].

Oogenesis in *Drosophila* is a developmental context rich in post-transcriptional control of gene expression [2]. Not surprisingly, small RNAs are active in this setting. The most extensive evidence is available for the rasiRNA pathway, for which the Argonaute proteins are Piwi, Aubergine (Aub) and AGO3. The Piwi and Aub proteins have well established roles during oogenesis in controlling stem cell divisions and in the events leading to formation of the embryonic germ line cells, but the details of their modes of action were not well understood [3]–[5]. More recently, Piwi, Aub and AGO3 have been found to associate with rasiRNAs, and *piwi* and *aub* mutants (no *AGO3* mutants have been described) are defective in the rasiRNA dependent silencing of various retrotransposons [6]–[10]. A number of genes have been implicated in the production of rasiRNAs; mutation of any of these genes results in deregulation of the transposons [7], [11], [12].

siRNAs were the first small regulatory RNAs shown to be active during oogenesis, but with limitations on when they can function. Late stage oocytes do not support RNA interference. However, this pathway is activated during egg activation, and introduction of exogenous dsRNAs results in degradation of target mRNAs [13]. Early in oogenesis the RNA interference pathway alters expression of *oskar* (*osk*) mRNA, apparently via indirect effects on the cytoskeleton [14]. It is not known why this pathway is not active throughout all of oogenesis. AGO2, the Argonaute protein that acts in RISC, the RNP complex that mediates RNA interference [15], is present for all the stages of oogenesis when immunodetection is possible (i.e., prior to deposition of the vitelline membrane), but whether it is present in the late stage oocytes that do not support RNA interference is unknown [9], [16].

Indirect evidence suggests that there will be miRNA-dependent control of translation in the ovary. Multiple miRNAs are present at high levels in early stage embryos, when zygotic transcription has not yet begun, and so these should have been provided maternally as a consequence of synthesis in the ovary [17]. Furthermore, several miRNAs have been directly shown to be present in the ovary [6]. A second line of indirect evidence comes from characterization of *AGO1* mutants. AGO1 is the Argonaute protein that acts in the miRNP, the RNP complex that mediates miRNA-dependent translational control [15]. Mutation and overexpression of *AGO1* each affect, with opposite consequences, the fate of germline stem cells, leading to the proposal that the miRNA pathway acts in this process [18]. However, the roles of different AGO proteins can overlap [19]. The roles of AGO1 and AGO2 were defined in cultured *Drosophila* S2 cells [15], and evidence that a particular protein has one role in one tissue does not necessarily mean that it will do the same in all other settings. The possibility of miRNA control in the germ line cells of the ovary was also raised by a peculiar feature of *osk* mRNA regulation. Specifically, in certain mutants with drastically reduced levels of Osk protein, a large fraction of *osk* mRNA is present in polysomes [20]. Such a phenomenon – polysome association of mRNAs not directing the accumulation of the encoded protein – is a feature of some mRNAs under miRNA control [1], although other regulatory pathways have the same effect [21].

If miRNA-dependent translational control does operate in the ovary, then the site or sites at which the regulation occurs will be of interest, and may provide information about possible mechanisms. At present, the mechanism of miRNA-dependent translational repression is a subject of controversy. Some studies have pointed to miRNAs intervening after the initiation of translation, which, as noted above, results in association with polysomes without protein accumulation [22]–[25]. Other analyses have revealed an effect of miRNAs on initiation of translation [26]–[29]. The latter option fits well with observed sites of miRNA regulation, in P bodies. P bodies were initially identified, in yeast, as RNPs containing mRNAs destined for degradation [30], [31]. More recently, mammalian P bodies have been found to contain mRNAs under miRNA control, as well as AGO proteins and other proteins required for the action of miRNAs [26], [32], [33]. Furthermore, in cultured *Drosophila* cells P body formation has been shown to be a consequence of RNA-mediated gene silencing, whether by *AGO1*- or *AGO2*-dependent pathways [34]. P bodies lack ribosomes [31], and so an mRNA under miRNA repression could not be in P bodies if it is associated with polysomes. In the germline cells of the *Drosophila* ovary there appear to be no conventional P bodies, but instead a higher order structure known as sponge bodies. Sponge bodies were first described as cytoplasmic sites at which the Exuperantia (Exu) protein, which acts in mRNA localization, is highly concentrated [35]. Subsequently, a number of other proteins with roles in post-transcriptional control of gene expression have been shown to colocalize with Exu [36]–[42]. Notably, the sponge body proteins include homologs of multiple P body components, and sponge bodies are largely devoid of ribosomes [35]. Sponge bodies and P bodies do not appear to be equivalent, as sponge bodies include cisternae and vesicles while no membrane is found in the P body-like GW182 bodies (there have been no ultrastructural studies of P bodies)[35], [43]. However, it does seem likely that sponge bodies represent a membrane-based framework upon which RNPs similar or equivalent to P bodies are positioned. By analogy to mammalian cells and to cultured *Drosophila* cells, these sponge body RNPs would be an expected site of miRNA action.

Here we directly test for miRNA-dependent translational repression in the ovary, and find that it does occur. We also show that there is no detectable concentration of repressed mRNAs with sponge bodies. Instead, the regulated mRNAs are present in numerous very small cytoplasmic particles. Both AGO1 and AGO2 (or GFP fusions to these proteins) appear in similar small particles. These results do not rule out any specific model for the mechanism by which miRNAs repress translation in the ovary, but do allow for translational inhibitory mechanisms that act after initiation of translation.

## Results

**Figure 1:**
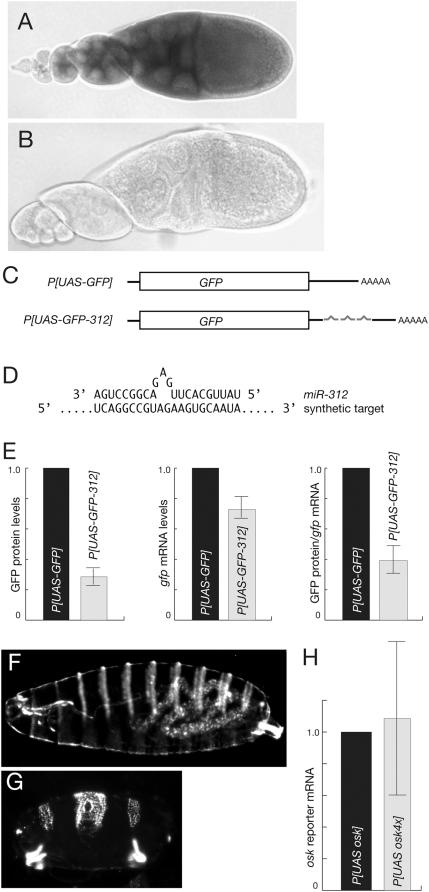
Expression and activity of *miR-312* in the ovary. A and B. In situ hybridization with locked nucleic acid probes. The probe in A is complementary to *miR-312*, while the probe in B has a scrambled sequence. The *miR-312* hybridization signal appears throughout these stages of oogenesis (levels are very low at the earliest stages of oogenesis, at the extreme left. C. *GFP* reporter transgenes to detect *miR-312* activity. The diagrams show the transgene mRNAs (not to scale). Transgenes with the *osk* coding region as the reporter are essentially the same, with replacement of the coding regions, except that the *GFP* reporter has 6 copies of the *miR-312* binding site while the *osk* reporter has 4 copies. D. Sequence of a single copy of the *miR-312* synthetic binding site, shown as it would base pair to *miR-312*. E. Translational repression of the *UAS-GFP-312* reporter transgene. GFP protein and mRNA levels were measured by quantitative western blotting and quantitative real time PCR, respectively, with levels from the *UAS-GFP* transgene normalized to 1. Averages from 3 or more experiments are shown, with standard deviations indicated. Normalization for mRNA levels, at right, reveals the level of translational repression per unit of mRNA. Note that the regulated and unregulated mRNAs are expressed from different transgenes and that any effect of the *miR-312* binding sites on mRNA levels can not be addressed in this analysis. If the presence of the *miR-312* binding sites reduces mRNA levels, as is common in cases of miRNA regulation, then the effective degree of negative regulation would be even greater. F. Wild type cuticle of an embryo from females expressing *UAS-osk-312*. G. Bicaudal cuticle of an embryo from females expressing *UAS-osk*. H. Levels of mRNA from *osk* reporter transgenes, measured by quantitative real time PCR and normalized to 1 for the unregulated *UAS-osk* transgene mRNA. Averages from 3 or more experiments are shown, with standard deviations indicated.

To test for miRNA activity in the *Drosophila* ovary we focused on *miR-312*. Northern blot analysis of miRNAs during embryogenesis has revealed that *miR-312* is present at the highest levels in 0–1 hour embryos, suggesting that it is expressed during oogenesis [17]. Indeed, *miR-312* expression in the ovary has been detected by a PCR assay [44]. We confirmed the ovarian expression of *miR-312* by in situ hybridization. A Locked Nucleic Acid probe for *miR-312* reveals the miRNA to be present throughout the ovary, in both germ line cells (nurse cells and the oocyte) and somatic follicle cells (Fig. 1A). By contrast, a probe with a scrambled sequence shows no hybridization (Fig. 1B).

To monitor potential *miR-312* repressive activity, a GFP reporter assay was developed. Six tandem copies of a synthetic *miR-312* binding site were added to the 3′ UTR of a *UAS-GFP* transgene (Fig. 1C). The binding sites were designed to allow incomplete base pairing with *miR-312*, such that the 5′ ‘seed’ and 3′ regions of the miRNA would be fully base paired, with a central unpaired bulge (Fig. 1D). Interrupted base pairing of this type usually leads to translational repression by miRNAs, rather than RNA degradation [45]–[47]. Multiple copies of the *miR-312* binding sites were used to increase the probability of efficient *miR-312* binding. Although no confirmed targets of *miR-312* action have been identified, the ovarian *kelch* mRNA is a candidate with 4 predicted *miR-312* binding sites [48]. Transcription of the reporter transgene relies on the UAS component, which responds to the GAL4 transcriptional activator [49], [50]. Following expression of the reporter (*UAS-GFP-312*) and control (*UAS-GFP*) transgenes in the germline cells of the ovary using the *matalpha4-GAL-VP16* driver, GFP protein and mRNA levels were measured by quantitative western blot analysis and quantitative real time PCR (RT-PCR). The transgene with *miR-312* sites produces significantly less GFP than the control transgene (Fig. 1E). This difference can be attributed in part, but not in whole, to unequal mRNA levels: the mRNA with *miR-312* targets is present at 70% of the level of the control transgene (Fig. 1E). Normalization for the different mRNA levels reveals that the *GFP -312* mRNA is about half as active as the control *GFP* mRNA in production of GFP protein (Fig. 1E). Thus, the presence of the *miR-312* target sites in the *GFP-312* mRNA confers translational repression.

As an additional assay of miRNA activity, a second set of transgenes was constructed and tested. The *UAS-osk* control transgene is similar to *UAS-GFP*, except that the coding region is now from an *osk* cDNA (with none of the *osk* 3′ UTR). *UAS-osk-312* differs from *UAS-osk* by the addition of four tandem copies of the synthetic *miR-312* binding site. When expressed in the ovary using the *matalpha4-GAL-VP16* driver, the *UAS-osk* control transgene produces ectopic Osk, which disrupts anterior embryonic body patterning resulting in a very high frequency of bicaudal embryos (96%; n = 131)(Fig. 1F). In contrast, expression of the *UAS-osk-312* transgene with the *miR-312* binding sites has no significant effect on embryonic patterning: the vast majority of embryos (94%; n = 373) appear wild type (Fig. 1G). Both transgene mRNAs are present at similar levels (Fig. 1H). Thus, in each of two assays the addition of *miR-312* binding sites to an mRNA leads to its translational repression.

**Figure 2:**
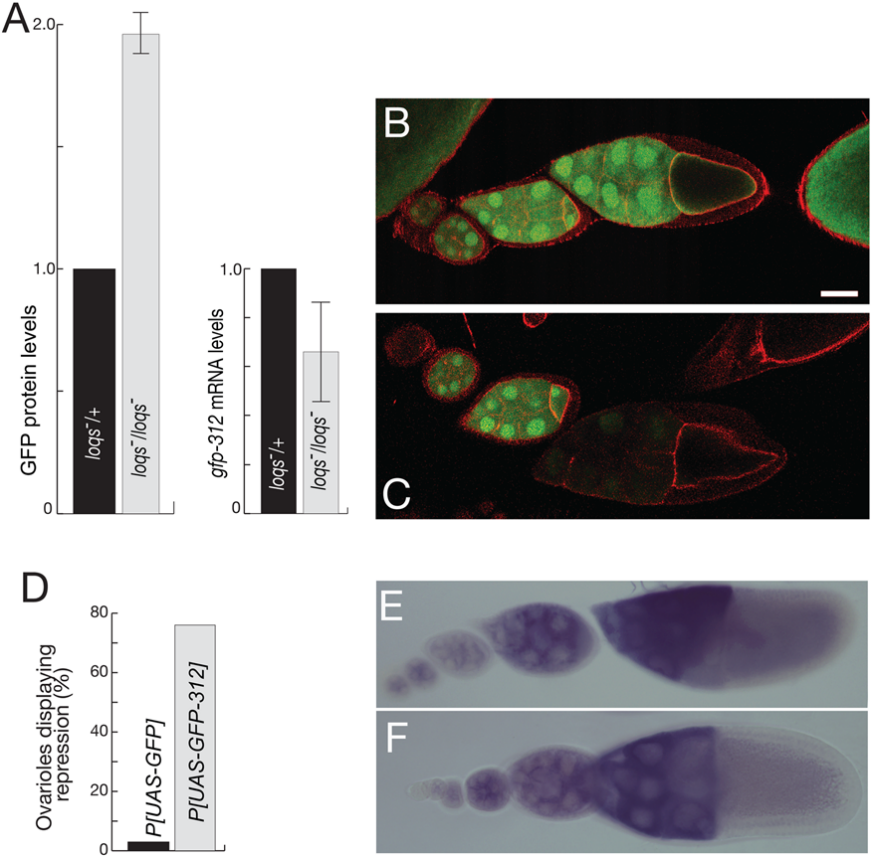
Translational repression of *GFP-312* mRNA requires miRNA production. A. Quantitation of protein and mRNA levels from the *UAS-GFP-312* transgene in females heterozygous or homozygous for *loqs^f00791^*. Levels were determined as in Figure 1. B and C. GFP in ovarioles expressing the *UAS-GFP* (B) or *UAS-GFP-312* (C) transgenes. GFP is in green, and phalloidin staining in red. GFP signal intensity at early stages of oogenesis (to the left in the panels) is typically lower for the *UAS-GFP-312* transgene, but was adjusted to show a similar intensity as for *UAS-GFP* at those stages; this better reveals the extent of the difference between GFP levels for the two transgenes at later stages of oogenesis (at right). D. Quantitation of early/late stage GFP levels in individual ovarioles. Fluorescence intensity was measured in stage 7 or 8 and 10 egg chambers of individual ovarioles. If the ratio of fluorescence in stage 7/8 to stage 10 was greater than 1.5, then the ovariole was considered to show repression. E and F. In situ hybridization detection of *GFP* (E) and *GFP-312* (F) mRNAs.

Translational repression of the reporter mRNAs could be due to the action of miRNAs, or could arise in some other manner because of the addition to the reporter mRNA of the sequences that make up the *miR-312* binding sites. To distinguish between these options we asked if miRNAs are required for the observed repression of the GFP reporter mRNA. Loquacious (Loqs) protein acts in processing pre-miRNAs, and *loqs* mutants are defective in this process [51]. In *loqs* mutant ovaries the level of GFP produced from the *UAS-GFP-312* transgene was elevated about 2× relative to *loqs/+* heterozygotes, while mRNA levels did not show a corresponding increase (Fig. 2A). This change in protein level corresponds well to the observed level of translational repression, and demonstrates that repression is dependent on miRNAs.

GFP levels in individual egg chambers were also examined by confocal microscopy. Comparison of ovaries expressing either the *GFP* or *GFP-312* mRNAs reveals two differences. First, the overall GFP level was typically lower from the mRNA with the *miR-312* targets, consistent with the quantitative western blot analysis. Second, GFP levels dropped at later stages for the *GFP-312* mRNA, but not for the *GFP* mRNA (Fig. 2B,C). To quantify this effect GFP levels were measured at different stages of oogenesis within individual ovarioles: at stage 7/8 (before the reduction) and at stage 10. Of the ovarioles expressing *GFP-312* mRNA, greater than 75% showed at least a 1.5 fold reduction in GFP at the later stage, while less than 5% of ovarioles expressing *GFP* mRNA showed such a reduction (Fig. 2D).

Although the quantitative measures of mRNA and protein levels for the reporter transgenes clearly demonstrate an effect at the level of protein accumulation, it remains possible that *miR-312* is also affecting the stability of the reporter mRNA. Because the reporter and control mRNAs are expressed from different transgenes, the site of transgene insertion might influence transcription (transgenes under UAS/GAL4 control do show some line-to-line variability in expression levels). Thus we cannot readily determine if the differences in levels for the *GFP-312* and *GFP* mRNAs are due to the action of miRNAs. However, it is simple to determine if the late stage reduction in GFP translated from the *GFP-312* mRNA is accompanied by a reduction in transcript levels. For both *GFP* and *GFP-312* mRNAs there is a substantial increase in mRNA levels later in oogenesis, rather than a decrease (Fig. 2E,F), consistent with the known activity of the GAL4 driver used for expression [52]. Why the *GFP-312* mRNA is more effectively repressed at later stages of oogenesis is uncertain. The levels of *miR-312* RNA appear to increase during this period (Fig. 1A), but attempts to use fluorescent-based in situ hybridization to obtain more quantitative data that would address this possibility have not been successful.

### Sites of miRNA activity in the ovary

Having demonstrated that miRNA-dependent translational repression does occur in the ovary, we wished to ask if the repressed mRNAs and factors involved in repression are concentrated in sponge bodies.

**Figure 3:**
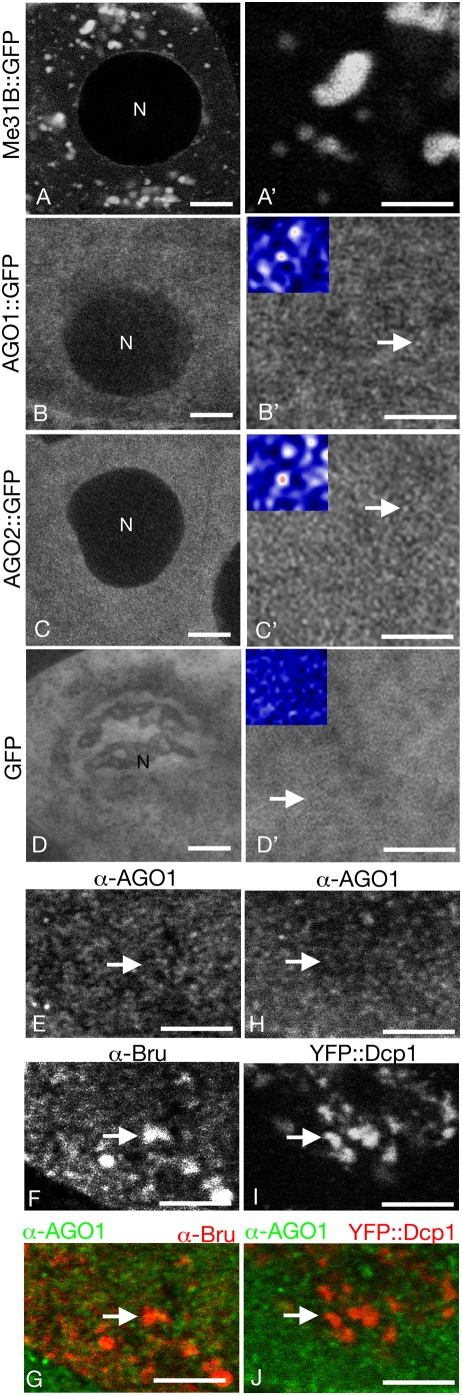
Subcellular distribution of AGO and AGO::GFP proteins in the ovary. Panels A–D show the distributions of GFP fusion proteins in the nurse cells of live stage 9 egg chambers, with higher magnification images in A'–D'. Scale bars are 10 µm in A–D and 5 µm in A'–D'. A, Me31B::GFP; B, AGO1::GFP; C, AGO2::GFP; and D, GFP. The lower resolution images (with the nuclei marked as N) demonstrate the absence of any detectable concentration of either of the AGO::GFP fusion proteins in localized regions of the cytoplasm, as would be the case if they are enriched in sponge bodies. Sponge body distribution is shown by Me31B::GFP. The higher resolution images reveal that both AGO::GFP fusion proteins are distributed in many small puncta that are fairly evenly spread throughout the cytoplasm (examples of individual puncta are indicated by arrows). By contrast, GFP alone (D') is more uniform and lacks puncta of the size seen for the fusion proteins. The insets in B'–D' are higher resolution images of the regions in B'–D' indicated by arrows. The representation of each fluorescence value was changed in ImageJ (NIH) from a black and white scale (look up table; LUT) to the Union Jack LUT which represents the lowest fluorescence as black and progressively higher fluorescence levels are blue, white, and then red. AGO1::GFP and AGO2::GFP fluorescence is concentrated in puncta (appearing white and red in the insets in B',C') that are not observed for GFP (inset in D). Panels E–G show a portion of a nurse cell of a fixed stage 9 egg chamber probed with antibodies to detect AGO1 (E) and Bru (F), or both (G; AGO1 in green, Bru in red). Panels H–I are similar, with detection of AGO1 (H), dDcp1::YFP (I) or both (J; AGO1 in green, dDCP1::YFP). The AGO1 distribution is particulate, just as for the AGO1::GFP fusion protein, and shows no enrichment in sponge bodies, for which Bru and dDcp1::YFP are markers ([41], [55]). Scale bars are 5 µm.

In one line of experiments the distributions of Argonaute family members were monitored, either by live imaging of GFP fusion proteins or by immunodetection in fixed samples. For live imaging a Me31B::GFP fusion protein was used to mark sponge bodies, and AGO1::GFP and AGO2::GFP distributions were evaluated. In other tissues AGO1 is primarily responsible for miRNA-mediated translational repression, while AGO2 acts in RNA interference [15]. In the ovary both AGO proteins are predominantly cytoplasmic, with much lower levels in the nuclei. This pattern is strikingly distinct from the characteristic sponge body distribution seen with the Me31B::GFP marker. Sponge bodies are also cytoplasmic, but are concentrated in discrete large domains (Fig. 3A).

At higher magnification, the distribution of both AGO1::GFP and AGO2::GFP can be seen to be punctate, with the proteins appearing in many small foci (Fig. 3B,C). This distribution is not characteristic of GFP fusion proteins [53]. Moreover, the distribution is distinctly different from that of GFP alone, which is more uniform in the cytoplasm (Fig. 3D). As a quantitative measure of the difference between the patterns of GFP and the AGO::GFP fusion protein distributions, we evaluated the range of fluorescence intensities. A uniform protein distribution in a given field should show a narrow range of fluorescence intensity. In contrast, a protein that is concentrated in particles should show a broader range of intensities: low outside of the particles, and high in the particles. Fluorescence intensities were assigned colors in a look up table (LUT)(see Fig. 3 legend). The variation in fluorescence intensity is much greater for AGO1::GFP and AGO2::GFP than for GFP (Fig. 3B'–C'). This variation confirms that both AGO fusion proteins are concentrated at many small foci. In a direct comparison of protein distributions by immunostaining of fixed egg chambers, AGO1 was detected in tiny foci similar in number and distribution to those detected with AGO1::GFP in live samples (Fig. 3E), while Bruno (Bru; a sponge body component; [54], [55]) was largely non-overlapping and predominantly in the much larger sponge bodies (Fig. 3F,G). Similarly, the *Drosophila* ortholog of Dcp1, a component of P bodies in other organisms [56], [57] and of sponge bodies in the *Drosophila* ovary [41], was found in sponge bodies but not in the AGO1-positive puncta (Fig. 3I,J).

Knowledge of the AGO protein distribution in the egg chamber provides useful but nevertheless limited information about the sites of miRNA mediated repression. The absence of any enrichment of AGO1 and AGO2 proteins (or GFP fusions of these proteins) in sponge bodies suggests that sponge bodies are not the primary destination for mRNAs under miRNA control. The many small foci of AGO proteins in the cytoplasm are good candidates for sites of repression. However, it is also possible that the lower level of more uniformly distributed AGO proteins comprise the fraction actively engaged in regulation. We therefore took another approach to more directly monitor sites of miRNA action in the ovary.

**Figure 4:**
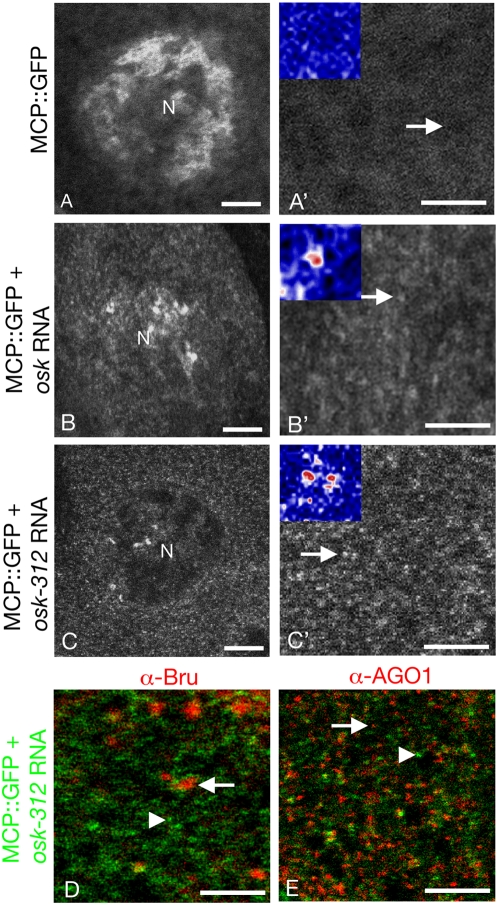
Sites of miRNA action in the ovary. Panels A–C show the distribution of MCP::GFP in nurse cells of live stage 10A egg chambers, with higher magnification images of cytoplasmic regions in A'–C'. Scale bars are 10 µm in A–C and 5 µm in A'–C'. A–C differ with respect to which reporter mRNAs with MS2 binding sites are present: A, no reporter mRNAs; B, reporter mRNAs lacking *miR-312* binding sites; and C, reporter mRNAs with *miR-312* binding sites. The cytoplasmic MCP::GFP distributions were the same at earlier stages of oogenesis. Note that the size of the particles is similar to those marked with the AGO::GFP fusion proteins in Fig. 3, but the number of particles seen in each field is lower. The insets in A'–C' are a higher resolution image of the regions in A'–C' indicated by arrows, with Union Jack LUTs as described in the legend to Fig. 3. MS2::GFP in the presence of the *osk* reporter mRNAs, with or without *miR-312* binding sites, is cosncentrated in puncta (appearing white and red in the insets in B',C') that are not observed with MS2::GFP alone (inset in A'). Panels D and E show the distribution of MCP::GFP (green in D and E) tethered to the *osk-312* reporter transcripts, and either sponge bodies (revealed by anti-Bru; red in D) or AGO1 puncta (red in E). The sponge bodies and AGO1 puncta (arrows in D and E) do not colocalize with the tethered reporter transcripts (arrowheads in D and E). Scale bars are 5 µm in D and E.

Using a tethering assay, control mRNAs and mRNAs under miRNA repression were tracked in live samples. In this type of assay, a fusion protein consisting of an RNA binding domain and GFP is tethered to an mRNA bearing the appropriate RNA binding sites [58]. We used an MS2 coat protein::GFP (MCP::GFP) fusion protein previously shown to work in this assay in *Drosophila* egg chambers [59], together with *UAS-osk* reporters as described above (the *UAS-GFP* type of reporter transgene could not be used, as expression of GFP from the reporter would create a high background that would interfere with specific detection of the tethered MCP::GFP). In the absence of any transcripts with the MS2 binding sites, the MCP::GFP protein is largely nuclear (Fig. 4A) as it contains a nuclear localization signal (NLS). However, when reporter transcripts with MCP binding sites (with or without *miR-312* target sites) are also present, the MCP::GFP adopts a new distribution: it is now predominantly cytoplasmic (Fig. 4B,C). The shift to the cytoplasm presumably results from nuclear export of reporter mRNAs. Bound molecules of MCP::GFP are thus moved into the cytoplasm, reducing the fraction of MCP::GFP in the nuclei. The UAS/GAL4 expression system allows for high levels of transcription, and it is therefore not surprising that the reporter transcripts can influence the balance of MCP::GFP between the nucleus and cytoplasm.

Within the cytoplasm the tethered reporter mRNAs, both regulated and unregulated, are present in very small particles distributed in very similar patterns (Fig. 4A,B). In contrast, MCP::GFP in the absence of reporter transcripts is more evenly distributed (Fig. 4A). The reporter transcripts do not appear in large puncta, indicating that they, like the AGO proteins, are not enriched in sponge bodies. As a more direct demonstration of this property, the sponge body component Bru was detected simultaneously with MCP::GFP in fixed samples of these ovaries. No concentration of the tethered reporter mRNAs in the Bru-containing sponge bodies was detected (Fig. 4D).

The patterns in which AGO proteins and regulated and unregulated reporter transcripts are distributed are indistinguishable. This similarity could simply indicate that each molecule is found in many small RNP particles that are dispersed throughout the cytoplasm. If the foci of transcripts under miRNA regulation do correspond to sites of repression, then they might be expected to be coincident with the foci of AGO1 protein. We tested this prediction by double labeling. Notably, there is very little overlap in the two types of foci (Fig. 4E).

## Discussion

Evidence presented here addresses the possibility that miRNA dependent control of gene expression occurs in the *Drosophila* ovary. The miRNA *miR-312* is expressed during oogenesis, and addition of synthetic *miR-312* binding sites to either of two different reporter mRNAs reduces their activities. This effect is dependent on the ability of the ovary to synthesize miRNAs. Collectively, these results make a compelling case for activity of miRNAs in the ovary. Furthermore, the data show that the stages when miRNA dependent regulation occurs include the mid to late stages of oogenesis, when post-transcriptional control of gene expression plays a crucial role in the events that lead to patterning of the embryo. Thus, miRNAs have the potential to contribute to this process.

Although we have focused only on a single miRNA, many of the known miRNAs are present at high levels in early stage embryos that have not initiated zygotic transcription [17], and these miRNAs should also be present in the germ line cells of the ovary. Our evidence shows that the miRNA machinery is competent for function during oogenesis, and it would be very unlikely that only *miR-312* can make use of that machinery. Thus, there is every expectation that in oogenesis numerous miRNAs are actively engaged in regulation and many mRNAs are regulated by miRNAs.

To address the question of where in the ovary miRNA-mediated translational repression occurs, we monitored the distribution of AGO proteins and reporter mRNAs under miRNA control. Using high resolution confocal microscopy and detection either by immunofluorescence in fixed ovaries or live imaging of an AGO1::GFP fusion, we find that AGO1 is present in many small foci rather than being uniformly dispersed throughout the cytoplasm. A similar pattern is observed for reporter mRNAs detected by indirect labeling with tethered GFP, independent of whether they are under miRNA control. The AGO1-containing foci are distinct from those containing the regulated reporter mRNAs. Thus, the significance of the small foci remains uncertain.

The main conclusion of this portion of our work is that sponge bodies are not the primary sites of miRNA activity. Notably, neither AGO1 nor the repressed mRNAs display any detectable concentration in sponge bodies (see also [16]). Sponge bodies are similar to P bodies, with many shared components [36]–[42]. In some other cell types P bodies are enriched in miRNP components, and can readily be seen as bright foci on a darker background when either miRNP protein components or regulated mRNAs are detected with fluorescent labels [26], [33], [60]. This enrichment initially suggested that a major fraction of the miRNP components are in P bodies. However, subsequent quantitative analyses revealed that only a very small fraction (less than 2%) of the miRNP AGO protein is in the P bodies, and that the vast majority of the protein is in the cytoplasm but diffuse and therefore more difficult to detect [61]. By way of comparison, then, our failure to detect any enrichment of AGO1 or transcripts under miRNA control in sponge bodies indicates that the fraction present must be substantially less than the 2% of the cited example; in effect, there can be essentially no enrichment at all.

The distribution of miRNPs within the cell is relevant to the mechanism by which translation is repressed by miRNAs. P bodies lack ribosomes, and the presence of certain miRNPs and their regulated mRNAs within P bodies indicates that these mRNAs are not undergoing translation. This fits well with some models for miRNA-dependent repression, in which the initiation of translation is inhibited [26]–[29]. There is also evidence for regulation by miRNAs after initiation of translation, with the repressed mRNAs being associated with polysomes [22]–[25]. Obviously, such a mechanism could not apply for the mRNAs found in P bodies. Consequently, any enrichment of miRNPs in sponge bodies (whose similarity with P bodies includes an almost complete lack of ribosomes [35]), would be consistent with repression blocking initiation of translation. The observed absence of such an enrichment raises the possibility that whatever mechanism delivers miRNPs to the P bodies is not operating in the germline cells of the ovary (where the sponge bodies are found), at least for the developmental stages examined. While this does not rule out miRNA-dependent translational repression at the level of initiation, it does leave open the possibility that action of miRNAs after initiation of translation may be more prominent in this setting than in cells with conventional P bodies.

## Materials and Methods

### Plasmids

*UAS-GFP* (p8508) is pUASp [50] to which *mGFP6* [61] has been introduced as an Asp718 fragment into the Asp718 site. The *mGFP6* Asp718 fragment begins with GGTACCCAATTCGTTAACAGATCCAAGGAGATATAACA prior to the *mGFP6* start codon, and ends with CTCGAGGGTACC after the *mGFP6* stop codon. A synthetic single *miR-312* binding site cassette was constructed by PCR and had the sequence 5′TCTAGATCAGGCCGTAGAAGTGCAATACTAGT 3′. This cassette, which includes XbaI and SpeI sites at the 5′ and 3′ ends, was multimerized using the XbaI and SpeI sites. A 6× version was inserted into the XbaI site of *UAS-GFP* to make *UASp-GFP-312* (p8701). A 4× version of the *miR-312* binding sites was introduced into the XbaI site between the *osk* coding region and UASp vector 3′ UTR of *UAS-osk* [62] to make *UAS-osk-312* (p8619). *UAS-osk-312* was further modified by addition of 16 copies of the MS2 coat protein binding site to the 3′ UTR [57] to make *UAS-osk-312-MS2* (p8492). *UAS-osk* was modified in the same manner to make *UAS-osk-MS2* (p8516).

### Fly strains

*loqs^f00791^* flies were from Bill Theurkauf. Transgenes constructed in this study were injected by Genetic Services, Inc. GFP trap stocks were the following: *AGO1^CA06914^*; *AGO2^CA07002^*; and *ME31B::GFP^ZCL1796^* [53], [64]. The latter GFP trap was recovered from stock ZCL1796, which was described as having an insertion that tags CG3634 on the third chromosome. Molecular characterization of this stock revealed that the GFP fluorescence from this line was entirely from a second GFP trap insertion in the *me31B* gene. *MS2::GFP* was from E. Gavis. The expression of UAS transgenes was driven by the *matalpha4-GAL-VP16* driver [52]. YFP::dDcp1 was from Tze-Bin Chou [41].

### Real-time RT PCR

RNA was isolated from dissected ovaries using Tri Reagent-LS according to the manufacturers instructions (Molecular Research Center). 2 µg of ovarian RNA was reverse transcribed using random primers and the High Capacity cDNA Reverse Transcription kit (Applied Biosystems). Real-time PCR was performed using the 7900HT Sequence Detector and the Power SYBR Green PCR Master Mix (Applied Biosystems). mRNA levels were determined by relative quantitation with a standard curve and normalized to *RpL32* mRNA. All experiments were performed at least 3 times. Primers to amplify cDNAs were the following: *GFP*, TTTTCGTTGGGATCTTTCGAA and ACGGCGGCGTGCAAC; *RpL32*, GCGCACCAAGCACTTCATC and GACGCACTCTGTTGTCGATACC; *osk*, GCGTTAGGTCCTGTTCATTGGT and GCCATCGCTTGGAGGAAAG.

### Quantitative western analysis

Ovaries were collected on ice and homogenized in SDS-PAGE loading buffer with protease inhibitors (5 mM benzamidine and 10 µM PMSF). Samples were boiled for 5 minutes and loaded on a SDS-PAGE gel. Western blots were performed using the LI-COR detection system according to the manufacturers instructions and imaged using a Odyssey Infrared Imaging System (LI-COR Biosciences). GFP protein levels were normalized relative to ß-tubulin. All experiments were performed at least 3 times. Anti-GFP antibodies were prepared by Josman Laboratories. ß-tubulin was detected with monoclonal antibody E7 from the Developmental Studies Hybridoma Bank.

### In-situ hybridization

To detect *miR-312*, a digoxigenin labeled Locked Nucleic Acid probe (TATTGCACTTGAGACGGCCTGA)(Exiqon) was used according to the manufacturers instructions with annealing temperature of 55°C. The sequence of the scrambled control probe is TTCACAATGCGTTATCGGATGT. To detect *GFP* mRNA, an antisense probe was prepared by in vitro transcription and labeled with digoxigenin. Hybridization and detection were performed as described [64] except that the first wash following hybridization was raised 3 degrees C (relative to the hybridization temperature) to reduce background.

### Immunofluorescence and microscopy

Ovaries were fixed, stained and imaged as described [63], except for the analysis of Fig. 2, in which ovaries were fixed as described [4] and stained with AlexaFluor 594 phalloidin (Molecular Probes). The area based analysis function of the Leica Confocal Software was used to measure the mean GFP fluorescence in the nurse cell cytoplasm. Fluorescence in regions of the same size was measured in three nurse cells from each egg chamber. Antibodies were used at the following dilutions: rabbit anti-AGO1 ab5070 (Abcam), 1/100; rat anti-Bru, 1/500. Live imaging of GFP was performed as described previously [63].
